# *EGFR* mutation detection in circulating cell-free DNA of lung adenocarcinoma patients: analysis of LUX-Lung 3 and 6

**DOI:** 10.1038/bjc.2016.420

**Published:** 2016-12-22

**Authors:** Yi-Long Wu, Lecia V Sequist, Cheng-Ping Hu, Jifeng Feng, Shun Lu, Yunchao Huang, Wei Li, Mei Hou, Martin Schuler, Tony Mok, Nobuyuki Yamamoto, Kenneth O'Byrne, Vera Hirsh, Neil Gibson, Dan Massey, Miyoung Kim, James Chih-Hsin Yang

**Affiliations:** 1 Guangdong Lung Cancer Institute, Guangdong General Hospital and Guangdong Academy of Medical Sciences, 106 Zhongshan 2nd Road, Guangzhou 510080, China; 2 Massachusetts General Hospital and Harvard Medical School, 55 Fruit Street, Boston, MA 02114, USA; 3 Xiangya Hospital, Central South University, 87 Xiangya Road, Changsha, Hunan 410018, China; 4 Jiangsu Provincial Tumor Hospital, 42 Baiziting, Xuanwu, Nanjing, Jiangsu 210009, China; 5 Shanghai Lung Tumor Clinical Medical Center, Shanghai Chest Hospital, Shanghai Jiao Tong University, 241 Huaihai West Road, Xuhui, Shanghai 200030, China; 6 Yunnan Tumor Hospital (The Third Affiliated Hospital of Kunming Medical University), Xingjie Alley, Xishan, Kunming, Yunnan, China; 7 Cancer Center, First Hospital of Jilin University, 71 Xinmin Street, Changchun, 130021, China; 8 West China Hospital, Sichuan University, 24 South Section 1, Yihuan Road, Chengdu, 610065, China; 9 West German Cancer Center, University Duisburg-Essen, Hufelandstraße 55, Essen 45147, Germany; 10 German Cancer Consortium (DKTK), Partner Site University Hospital Essen, Essen 45147, Germany; 11 The Chinese University of Hong Kong, Shatin, NT, Hong Kong SAR, China; 12 Wakayama Medical University, 811-1 Kimiidera, Wakayama, Wakayama Prefecture 641-8509, Japan; 13 Princess Alexandra Hospital and Queensland University of Technology, 199 Ipswich Road, Woolloongabba, QLD 4102, Australia; 14 McGill University, 845 Rue Sherbrooke O, Montréal, QC H3A 0G4, Canada; 15 Boehringer Ingelheim Pharma GmbH & Co. KG, Birkendorfer Street 65, Biberach 88400, Germany; 16 Boehringer Ingelheim Ltd UK, Ellesfield Avenue, Bracknell, Berkshire RG12 8YS, UK; 17 Boehringer Ingelheim GmbH, Binger Street 173, Ingelheim 55216, Germany; 18 National Taiwan University Hospital, 7 Chung-Shan South Road, Taipei 100, Taiwan

**Keywords:** afatinib, circulating cell-free DNA, ErbB family blocker, *EGFR* mutation, non-small cell lung cancer

## Abstract

**Background:**

In the Phase III LUX-Lung 3/6 (LL3/LL6) trials in epidermal growth factor receptor (*EGFR*) mutation-positive lung adenocarcinoma patients, we evaluated feasibility of *EGFR* mutation detection using circulating cell-free DNA (cfDNA) and prognostic and predictive utility of cfDNA positivity (cfDNA+).

**Methods:**

Paired tumour and blood samples were prospectively collected from randomised patients. Mutations were detected using cfDNA from serum (LL3) or plasma (LL6) by a validated allele-specific quantitative real-time PCR kit.

**Results:**

*EGFR* mutation detection rates in cfDNA were 28.6% (serum) and 60.5% (plasma). Mutation detection in blood was associated with advanced disease characteristics, including higher performance score, number of metastatic sites and bone/liver metastases, and poorer prognosis. In patients with common *EGFR* mutations, afatinib improved progression-free survival *vs* chemotherapy in cfDNA+ (LL3: HR, 0.35; *P*=0.0009; LL6: HR, 0.25; *P*<0.0001) and cfDNA− (LL3: HR, 0.46; *P*<0.0001; LL6: HR, 0.12; *P*<0.0001) cohorts. A trend towards overall survival benefit with afatinib was observed in cfDNA+ patients.

**Conclusions:**

Plasma cfDNA is a promising alternative to biopsy for *EGFR* testing. Detectable mutation in blood was associated with more advanced disease and poorer prognosis. Afatinib improved outcomes in *EGFR* mutation-positive patients regardless of blood mutation status.

Traditionally, treatment of non-small cell lung cancer (NSCLC) has consisted of platinum-based chemotherapy. However, over the past decade, the discovery of frequent molecular alterations in NSCLC, particularly epidermal growth factor receptor (EGFR) mutations, has led to a new treatment paradigm that includes targeted agents (Novello *et al*, 2016). The reversible EGFR tyrosine kinase inhibitors (TKIs) erlotinib and gefitinib, as well as the irreversible ErbB family blocker, afatinib, are approved for the treatment of advanced *EGFR* mutation-positive NSCLC. In this era of targeted therapies, identification of patients (e.g., via *EGFR* mutation detection) who may derive benefit from these agents is a key factor for successful treatment (Olsen and Jorgensen, 2014).

In clinical practice, *EGFR* mutations are routinely detected using DNA extracted from primary or metastatic tumour tissue obtained during tumour biopsy or resection, which is typically formalin fixed and paraffin embedded (FFPE) (Ellison *et al*, 2013; Jung, 2013). However, efficient and robust mutation testing using these tissue samples can be challenging owing to small biopsy size or lack of sufficient tumour cells, inherent issues associated with FFPE archival tissue (e.g., poor DNA quality and tissue necrosis), and reluctance or inability of patients to undergo invasive biopsy (Ellison *et al*, 2013; Jung, 2013; Weber *et al*, 2014). As such, the development of simpler, minimally invasive assays to detect gene mutations may provide an attractive alternative to tumour biopsy for the identification of patients eligible for targeted treatments.

An emerging methodology to this end is the detection of mutations in circulating cell-free DNA (cfDNA) isolated from blood (i.e., liquid biopsy). Clinical studies in patients with NSCLC have explored the feasibility of *EGFR* mutation detection using cfDNA from serum and/or plasma samples, as well as the prognostic value and potential utility of *EGFR* mutation positivity by cfDNA to predict clinical outcomes to EGFR-targeted therapies (Kimura *et al*, 2006, 2007; Bai *et al*, 2009; He *et al*, 2009; Brevet *et al*, 2011; Lee *et al*, 2011; Goto *et al*, 2012; Mok *et al*, 2013; Li *et al*, 2014; Douillard *et al*, 2014a, 2014b; Mok *et al*, 2015). These studies suggest that detection of *EGFR* mutation in the blood is feasible and may be useful in the absence of available tumour biopsy. However, there is variability in the detection rates of *EGFR* mutation in the blood compared with the standard methodology using tumour tissue, and the correlation of blood-derived *EGFR* mutation positivity with specific patient characteristics or clinical outcomes remains uncertain.

This article describes the findings of two large, randomised Phase III trials (LUX-Lung 3 (LL3) and LUX-Lung 6 (LL6)), which compared the ErbB family blocker afatinib with standard platinum-doublet chemotherapy (cisplatin+pemetrexed in LL3; cisplatin+gemcitabine in LL6) in treatment-naive patients with advanced NSCLC harbouring *EGFR* mutations in their tumours (Sequist *et al*, 2013; Wu *et al*, 2014; Yang *et al*, 2015). Improvements in clinical outcomes with afatinib *vs* chemotherapy in both LL3 and LL6, particularly in patients with tumours harbouring common *EGFR* mutations (Del19/L858R), have been previously reported (Sequist *et al*, 2013; Wu *et al*, 2014; Yang *et al*, 2015). Both trials prospectively collected paired tumour and blood samples from patients for analysis of *EGFR* mutations. The current analysis evaluates the technical feasibility of detecting *EGFR* mutations in cfDNA from either serum (LL3) or plasma (LL6) and explores the association of clinical characteristics and outcomes with cfDNA-positive (cfDNA+) or -negative (cfDNA−) status in *EGFR* mutation-positive patients.

## Materials and Methods

### Study design and patients

Details of the LL3 and LL6 study designs and patient eligibility criteria have been previously published (Sequist *et al*, 2013; Wu *et al*, 2014). In brief, eligible patients in LL3 (*n*=345; recruited globally) and LL6 (*n*=364; recruited in China, South Korea, and Thailand) had pathologically confirmed and previously untreated stage IIIB/IV lung adenocarcinoma, an Eastern Cooperative Oncology Group (ECOG) performance status of 0 or 1, measurable disease according to Response Evaluation Criteria in Solid Tumors (RECIST) version 1.1 (Eisenhauer *et al*, 2009), and adequate organ function. Tumours were confirmed as *EGFR* mutation positive at screening based on central laboratory analysis of biopsy tissue using a validated test kit (Therascreen EGFR 29; Qiagen, Manchester, UK), as described in detail below.

In each study, patients were randomised (2 : 1) to receive oral afatinib (40 mg day^−1^) or up to six cycles of intravenous pemetrexed (500 mg m^−2^) plus cisplatin (75 mg m^−2^) once every 21 days in LL3 or gemcitabine (1000 mg m^−2^; days 1 and 8) plus cisplatin (75 mg m^−2^; day 1) every 21 days in LL6 (Sequist *et al*, 2013; Wu *et al*, 2014). Stratification parameters included *EGFR* mutation type (Del19/L858R/other) and race (Asian/non-Asian; LL3 only). Patients were treated until disease progression, death, unacceptable adverse events or withdrawal of consent for any reason.

The primary end point of each study was progression-free survival (PFS; by independent blinded review) (Sequist *et al*, 2013; Wu *et al*, 2014). Key secondary end points for both studies included objective response (complete response (CR) or partial response (PR)), disease control (CR, PR or stable disease), and overall survival (OS). Other secondary end points included patient-reported outcomes of disease symptoms and health-related quality of life and safety, which were previously published (Sequist *et al*, 2013; Wu *et al*, 2014).

### Study conduct

Each study was conducted in accordance with the Declaration of Helsinki, International Conference on Harmonisation good clinical practice, local laws, and applicable regulatory requirements and was approved by the institutional review board or independent ethics committee of each centre. An independent Data Monitoring Committee monitored conduct. All patients provided written, informed consent for participation in the studies and provision of tumour and blood samples. Trial registration IDs are: ClinicalTrials.gov NCT00949650 (LL3) and NCT01121393 (LL6).

### Blood/tumour sample collection and *EGFR* mutation detection

Tumour tissue from each patient was obtained at an initial diagnostic procedure for NSCLC and was paraffin embedded. Tumour samples for *EGFR* mutation detection consisted of at least five 10 *μ*m unstained sections mounted on a non-charged microscopic slide and containing at least 20% tumour tissue. In addition to the tumour tissue sample, a mandatory 9-ml blood sample was collected from each patient at the start of treatment (day 1, course 1) in both trials; however, different DNA extraction methodologies were used (see below for further details). The selection of the DNA extraction kit was based on the recommendation from the manufacturer at the time of each study conduct.

In both tumour tissue and paired serum/plasma samples, genotyping of *EGFR* mutations was conducted at a central laboratory using a validated allele-specific quantitative real-time PCR kit (Therascreen EGFR 29; Qiagen) designed to detect 29 *EGFR* mutations (19 deletions in exon 19 (collectively termed Del19), L858R, three insertions in exon 20 (collectively termed Ins20), L861Q, G719S, G719A, G719C (or G719X), T790M, and S768I) against a background of wild-type genomic DNA. Although a formal assessment of the amount of serum/plasma used for each sample was not conducted, it is important to note that the Therascreen EGFR 29 assay includes DNA-loading controls, which indicate when a DNA sample is too dilute or is non-amplifiable, and provides a means of performing quality control of the DNA during the assay.

### Extraction of DNA from serum

Following phlebotomy (9 ml of venous blood collected over ethylenediaminetetraacetic acid (EDTA)), serum was prepared and frozen prior to shipment to the central laboratory. DNA preparation from frozen serum samples (3 ml) was performed using the QIAamp DNA Blood Mini Kit (Qiagen) according to the manufacturer's instructions.

### Extraction of DNA from plasma

Following phlebotomy (9 ml of venous blood collected over EDTA), plasma was prepared and frozen prior to shipment to the central laboratory. The EDTA plasma sample was thawed at room temperature and then transferred to a 15-ml tube and centrifuged for 10 min at 16 000 × *g* in a fixed-angle rotor at+4 °C. Proteinase K solution (400 *μ*l) was added to a 50-ml tube. The clarified plasma supernatant was carefully separated from the sediment layer, and a maximum of 4 ml was added to the Proteinase K solution. The sample volume was then adjusted to 4 ml as required using phosphate-buffered saline. Buffer ACL (3.2 ml, containing carrier RNA) was added to the 50-ml tube and the solution was incubated at 60 °C for 30 min. The Proteinase K-digested sample was then further processed as per the QIAamp Circulating Nucleic Acid Kit manufacturer's instructions (Qiagen), and the final DNA samples were eluted in 100 ml of buffer AVE.

### Tumour assessments

Tumour assessments were performed by computed tomography or magnetic resonance imaging every 6 weeks for the first 48 weeks and then every 12 weeks thereafter until disease progression or start of new anticancer therapy (Sequist *et al*, 2013; Wu *et al*, 2014). Scans were reviewed by an independent central imaging group consisting of both radiologists and oncologists who were blinded to treatment assignments. Number of metastatic sites was determined by the count of unique sites of metastases across all organs with metastases. The sum of the longest diameters of lesions is the sum of the baseline target lesions identified by an independent review according to the procedures defined in RECIST version 1.1 (Eisenhauer *et al*, 2009).

### Statistical analyses

To formally define the performance of the EGFR Therascreen 29 assay, test results from a mixture of *EGFR* mutation-positive and -negative samples would be required to calculate the sensitivity, specificity, negative predictive value (NPV), and positive predictive value (PPV) of the assay. Per eligibility criteria in LL3 and LL6, only patients with *EGFR* mutation-positive tumours were included (i.e., all patients had tumour tissue that was positive for one or more *EGFR* mutation type). As such, calculations for sensitivity, specificity, PPV, and NPV in this analysis make use of test results for all seven mutation types within each individual patient.

For each study, logistic regression models were used to test for any association between baseline demographic and disease characteristics and the *EGFR* mutation test result from blood (cfDNA+ or cfDNA−). Separate efficacy analyses were performed on patients who were cfDNA+ or cfDNA− in each study. Comparisons of PFS and OS between treatment arms were made via a log-rank test. Cox proportional-hazard models were used to derive hazard ratios (HRs) and 95% confidence intervals (CIs) comparing the two treatment arms. Kaplan–Meier estimates were used to construct survival curves and calculate median PFS and OS. Logistic regression models were used to compare the rates of objective response and disease control between treatment groups. Additional Cox proportional-hazard models were used to assess whether the blood test result (cfDNA+ or cfDNA−) was prognostic for PFS or OS after adjusting for any effects of treatment. Further Cox proportional-hazard models were used, including the treatment-by-blood test result interaction term, in order to evaluate any evidence of heterogeneity. Efficacy analyses were restricted to those patients with an *EGFR* mutation blood test result whose tumour harboured a common *EGFR* mutation (Del19/L858R). Analyses were performed on all such patients and also for patients split by the type of mutation (Del19 or L858R) in the tumour. Efficacy data presented here are from the time point of the main OS analysis, when it was estimated that OS would be sufficiently mature (Yang *et al*, 2015). All analyses were *post hoc* and exploratory in nature.

## Results

### *EGFR* mutations in blood cfDNA and matched tumour tissue

**Figure 1:**
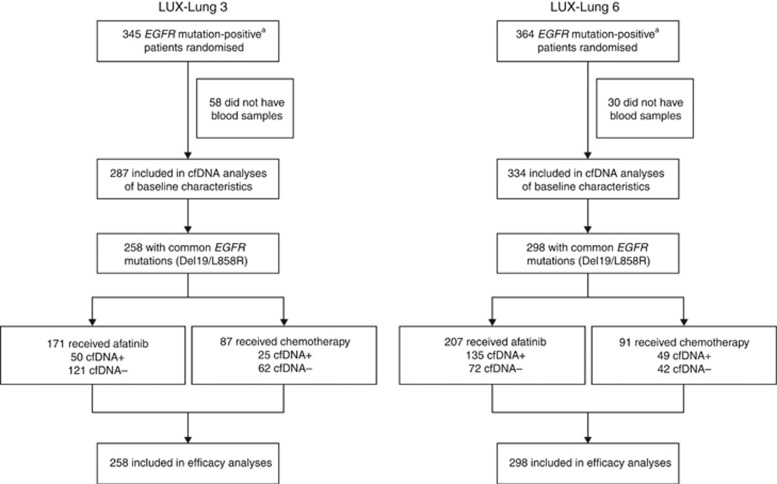
**Patient disposition in LUX-Lung 3 and LUX-Lung 6.** ^a^Patients who were *EGFR* mutation positive based on tissue biopsy at screening. Abbreviations: cfDNA=cell-free DNA; *EGFR*=epidermal growth factor receptor.

In LL3 and LL6, 287 (serum) and 334 (plasma) blood samples were analysed for the presence of *EGFR* mutations (Figure 1). The detection rates of *EGFR* mutation using cfDNA from blood samples compared with paired tumour samples were 28.6% (82 out of 287) in LL3 and 60.5% (202 out of 334) in LL6. Detection rates for individual *EGFR* mutations, including the common mutations Del19 and L858R, as well as various uncommon mutations, are shown in Table 1.

### Characteristics of patients with or without detectable *EGFR* mutation in the blood

Descriptive summaries supported by statistical tests of association showed that, in both trials, patients who were cfDNA+ for *EGFR* exhibited characteristics associated with more advanced disease compared with cfDNA− patients. cfDNA+ patients more frequently had a higher ECOG performance status (LL3: *P*=0.0029; LL6: *P*=0.0022), greater tumour burden (measured as the sum of the longest diameter of target lesions; approaching significance in LL3: *P*=0.061; LL6: *P*=0.0001), more metastatic sites (LL3: *P*=0.0003; LL6: *P*<0.0001), and metastases to the bone (LL3 and LL6: *P*<0.0001) and liver (LL3: *P*<0.0001; LL6: *P*=0.0003; Table 2). In terms of laboratory parameters, the cfDNA+ group also exhibited a general trend in higher laboratory values for alkaline phosphatase (ALP), lactate dehydrogenase (LDH), and white blood cell count (approaching significance for LL6), which are associated with metastatic spread to the bone and liver (2015). In addition, there was evidence in LL6 that female patients and those with a poorly/undifferentiated tumour grade were more likely to be cfDNA+ than cfDNA−. No notable differences were observed based on age, race, smoking status, disease stage, pleural metastases, brain metastases, or original *EGFR* mutation type in the tumour in either study.

### Efficacy analysis by cfDNA *EGFR* mutation detection status

Efficacy analyses were performed in NSCLC patients whose tumours harboured common *EGFR* mutations (Del19 or L885R) confirmed by tissue biopsy. Within this group of patients, comparisons between treatment arms (afatinib *vs* chemotherapy) were performed in those who were cfDNA+ or cfDNA− for any *EGFR* mutation based on their blood test result.

### Survival outcomes

**Figure 2:**
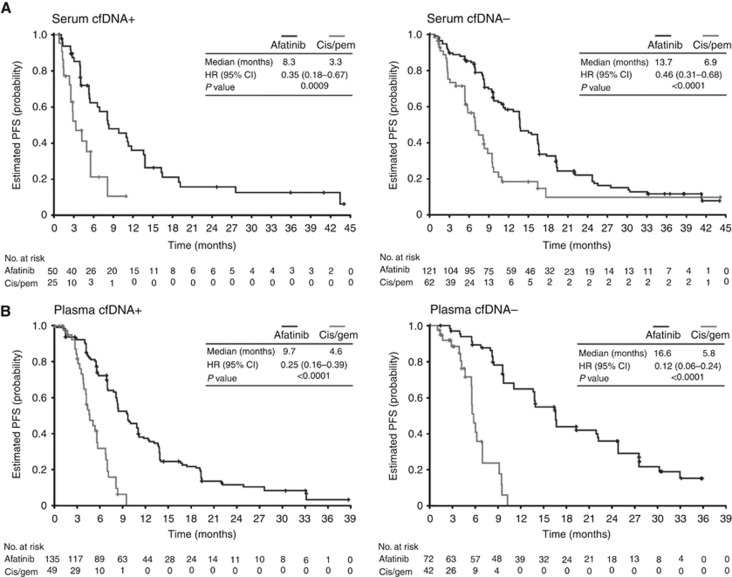
**Kaplan–Meier curves of PFS.** Kaplan–Meier curves of PFS according to the presence of an *EGFR* mutation by cfDNA analysis^a^ in patients with common *EGFR* mutations (Del19 or L858R; based on tumour biopsy) in (**A**) LUX-Lung 3 and (**B**) LUX-Lung 6. ^a^Patients who were *EGFR* Del19 or L858R mutation positive based on tissue biopsy were grouped according to whether any *EGFR* mutation was detected by cfDNA analysis (cfDNA+ or cfDNA−). Abbreviations: cfDNA=cell-free DNA; cis=cisplatin; *EGFR*=epidermal growth factor receptor; gem=gemcitabine; pem=pemetrexed; PFS=progression-free survival.

Afatinib significantly improved PFS *vs* chemotherapy in patients with common *EGFR* mutations in both the cfDNA+ (LL3: 8.3 *vs* 3.3 months; *P*=0.0009; LL6: 9.7 *vs* 4.6 months; *P*<0.0001) and the cfDNA−group (LL3: 13.7 *vs* 6.9 months; *P*<0.0001; LL6: 16.6 *vs* 5.8 months; *P*<0.0001; Figure 2). The PFS benefit of afatinib over chemotherapy was more pronounced in patients with Del19 *vs* L858R mutation-positive tumours (Table 3).

**Figure 3:**
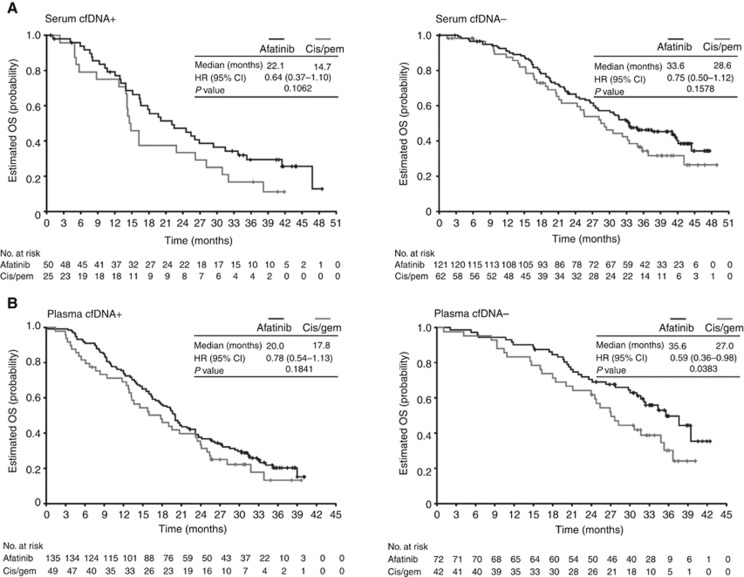
**Kaplan–Meier curves of OS.** Kaplan–Meier curves of OS according to the presence of an *EGFR* mutation by cfDNA analysis^a^ in patients with common *EGFR* mutations (Del19 or L858R; based on tumour biopsy) in (**A**) LUX-Lung 3 and (**B**) LUX-Lung 6. ^a^Patients who were *EGFR* Del19 or L858R mutation positive based on tissue biopsy were grouped according to whether any *EGFR* mutation was detected by cfDNA analysis (cfDNA+ or cfDNA−). Abbreviations: cfDNA=cell-free DNA; cis=cisplatin; *EGFR*=epidermal growth factor receptor; gem=gemcitabine; OS=overall survival; pem=pemetrexed.

A trend towards improvement in OS with afatinib *vs* chemotherapy was observed in patients who were cfDNA+ (LL3: 22.1 *vs* 14.7 months; LL6: 20.0 *vs* 17.8 months) or cfDNA− (LL3: 33.6 *vs* 28.6 months; LL6: 35.6 *vs* 27.0 months; Figure 3). In Del19 patients, a statistically significant improvement in OS with afatinib *vs* chemotherapy was observed in cfDNA+ patients in LL3 (29.4 *vs* 14.3 months; *P*=0.0372) and in cfDNA− patients in both trials (LL3: 33.3 *vs* 28.2 months; *P*=0.0247; LL6: 39.4 *vs* 21.1 months; *P*=0.0063; Table 3). OS was numerically improved in Del19 patients with a detectable *EGFR* mutation in plasma in LL6 (23.6 *vs* 14.6 months). No difference in OS with afatinib *vs* chemotherapy was observed in the L858R group for those with or without a detectable *EGFR* mutation in the blood (Table 3).

cfDNA+ patients had generally shorter PFS and OS in both treatment arms than cfDNA− patients (Supplementary Figures S1 and S2). Additional analyses confirmed the prognostic effect of a detectable *EGFR* mutation in the blood on PFS and OS; after adjusting for any effects of treatment, a consistent, statistically significant effect was observed (Table 3). There was no evidence to suggest that the treatment effect (afatinib *vs* chemotherapy) was different for cfDNA+ or cfDNA− patients, as observed by the non-significant interaction term *P* values (Table 3).

### Tumour response

In both cfDNA+ and cfDNA− patients, higher objective response rates (ORRs) based on independent review were observed in those treated with afatinib *vs* chemotherapy (cfDNA+ LL3: 62.0% *vs* 20.0% LL6: 69.6% *vs* 30.6% cfDNA− LL3: 62.8% *vs* 24.2% LL6: 69.4% *vs* 19.0%). Higher ORRs with afatinib *vs* chemotherapy in cfDNA+ patients (LL3: 78.0% *vs* 40.0% LL6: 76.3% *vs* 34.7%) and cfDNA− patients (LL3: 76.9% *vs* 45.2% LL6: 75.0% *vs* 33.3%) were also observed based on investigator assessment of tumour response.

## Discussion

The development of minimally invasive assays to detect gene mutations may provide a promising alternative to tumour biopsy, in the absence of available tissue samples, for identifying patients who would benefit from genotype-directed therapy such as *EGFR*-targeted agents. In the current analysis of NSCLC patients enrolled in the Phase III LL3 and LL6 trials, the *EGFR* mutation detection rates between paired tumour and blood samples suggest that the isolation of cfDNA from plasma using the QIAamp Circulating Nucleic Acid Kit (Qiagen) could be a potential alternative to tumour tissue for *EGFR* mutation testing in the absence of available tumour samples. Whether the difference in *EGFR* mutation detection rates between LL3 (28.6%) and LL6 (60.5%) in this analysis was attributable to the different DNA extraction kits used, the difference in DNA source (serum or plasma), or both is currently unclear. However, previous studies suggest that plasma may be a more reliable source than serum for tumour DNA owing to higher background levels of non-tumour wild-type DNA found in serum (Steinman, 1975; Board *et al*, 2008). In a recent analysis of *BRAF* mutation testing using amplification refractory mutation system (ARMS) methodology on cfDNA isolated from either serum or plasma collected from advanced melanoma patients, a greater amount of total cfDNA was isolated from serum *vs* plasma (using the QIAamp Circulating Nucleic Acid Kit (Qiagen) for each blood sample type); however, the proportion of tumour-derived mutant cfDNA was significantly greater in plasma than in serum (approximately 2 × higher) (Aung *et al*, 2014). Although total cfDNA isolated from serum *vs* plasma was not investigated in the current analysis, it is possible that the higher *EGFR* mutation detection rate observed in plasma *vs* serum may reflect a higher proportion of tumour-derived mutant cfDNA present in plasma.

In previous analyses, the detection rates for *EGFR* mutation in blood (plasma or serum) cfDNA compared with paired tumour tissue samples have ranged from 31% to 97% (Kimura *et al*, 2006, 2007; Moran *et al*, 2007; Bai *et al*, 2009; He *et al*, 2009; Kuang *et al*, 2009; Yung *et al*, 2009; Brevet *et al*, 2011; Goto *et al*, 2012; Liu *et al*, 2013; Zhao *et al*, 2013; Douillard *et al*, 2014b; Mok *et al*, 2015). This wide variation could be due to multiple factors, including differences in sample media (e.g., plasma *vs* serum), differences in DNA extraction and mutation detection methodologies (e.g., ARMS, digital PCR, or direct sequencing, among others), disease stage of patients (e.g., high cfDNA levels have been associated with more advanced disease; Lee *et al*, 2011), and the inclusion of patients with wild-type *EGFR*. Indeed, it is easier to establish the absence of an *EGFR* mutation in the blood than confirm its presence, suggesting that the inclusion of patients with wild-type *EGFR* may result in higher overall concordance rates with paired tissue samples.

In LL3 and LL6, *EGFR* mutation-positive patients who were cfDNA+ tended to have more advanced disease characteristics compared with cfDNA− patients, including higher ECOG performance scores, greater tumour burden, a higher number of metastatic sites, metastases to the bone and liver, and higher laboratory values (e.g., ALP and LDH), which are associated with metastatic spread to the bone/liver. This association may be a result of increased escape of tumour cells and DNA into the blood in patients with greater tumour burden and more metastatic sites. Furthermore, the more advanced baseline disease characteristics of *EGFR* cfDNA+ patients are likely to contribute to the poorer prognosis of these patients, irrespective of treatment with afatinib or chemotherapy. The correlation between the presence of mutant *EGFR* in the blood and the increased number of metastatic sites has been reported in a previous analysis (Lee *et al*, 2011). The higher proportion of females among cfDNA+ patients observed in LL6 is also consistent with some previous reports (Kimura *et al*, 2007; Jiang *et al*, 2011). Although some studies have demonstrated a correlation between cfDNA+ status and tumour histology, poor differentiation status, and non-smoking status (Kimura *et al*, 2007; Bai *et al*, 2009; Jiang *et al*, 2011; Zhao *et al*, 2013), such correlations were not observed in this report.

The therapeutic sensitivity of *EGFR* mutation-positive NSCLC to EGFR TKIs is well established (Yap and Popat, 2014). In this context, previous studies have demonstrated improved clinical outcomes in gefitinib-treated NSCLC patients with detectable *EGFR* mutations in serum (Kimura *et al*, 2006, 2007) and plasma (Bai *et al*, 2009) *vs* patients with NSCLC harbouring wild-type *EGFR*. The current study examined clinical outcomes in *EGFR* mutation-positive patients diagnosed by tumour biopsy according to their *EGFR* mutation status by cfDNA. In this analysis, clinical benefit was observed with afatinib- *vs* platinum-based chemotherapy whether or not the mutation could be found in the blood. Interestingly, cfDNA+ patients displayed less favourable PFS and OS, irrespective of treatment, compared with cfDNA− patients, despite similar ORRs in cfDNA+ and cfDNA− patients. Similar findings were observed in a recent study by Li *et al* (2014), in which shorter PFS with EGFR TKI treatment was reported in cfDNA+ *vs* cfDNA− NSCLC patients with *EGFR* mutation-positive tumours by biopsy. Subgroup analyses in the Phase III EURTAC trial in *EGFR* mutation-positive patients also demonstrated shorter PFS with erlotinib in cfDNA+ *vs* cfDNA− patients (Rosell *et al*, 2012). More recent analyses from the EURTAC trial demonstrated that, in patients with L858R mutations in the tumour, median OS was shorter for cfDNA+ patients compared with those who were cfDNA− (Karachaliou *et al*, 2015); these findings are consistent with results from our analysis. In contrast to our results, patients with tumour Del19 mutations who were also cfDNA+ had longer median OS than patients who were cfDNA−, and analysis of baseline disease characteristics did not show any significant differences between cfDNA+ and cfDNA− patients (Karachaliou *et al*, 2015). In recent analyses of the Phase III FASTACT-2 study, improved PFS with erlotinib over placebo was noted in patients who were cfDNA+ *vs* cfDNA− for *EGFR* mutations at baseline. However, for the baseline cfDNA+ subgroup, both PFS and OS were significantly worse for those who were cfDNA+ at cycle 3 of treatment compared with those who were cfDNA− at cycle 3 (Mok *et al*, 2015). In the Phase III First-SIGNAL study, high *EGFR* mutation positivity in plasma was associated with significantly shorter survival in gefitinib-treated patients with lung adenocarcinoma *vs* low *EGFR* mutation positivity (*P*=0.03) (Lee *et al*, 2011). Of note, high blood *EGFR* mutation positivity in the First-SIGNAL study was also associated with more aggressive baseline tumour characteristics (e.g., greater number of metastatic sites); Mok *et al* (2015) also hypothesised that the changes in outcomes with erlotinib may have been linked to increases in tumour burden or metastases.

To our knowledge, the current analysis of the LL3 and LL6 trials provides the largest cohort of data to date assessing both the feasibility of detecting *EGFR* mutation in the blood and the correlation of clinical characteristics and outcomes with cfDNA+ status. In this context, there are some limitations of the analyses that should be considered when interpreting the findings. First, blood samples were not collected from all screened patients in these studies, and the proportions of missing samples, while generally low, were different between LL3 and LL6, which may be a confounding factor in these analyses. Second, quantitative measurement of the extracted DNA was not performed; thus we were unable to determine whether there was a relationship between DNA quantity and test positivity. In addition, because all patients included in LL3 and LL6 had tumour tissue that was positive for one or more *EGFR* mutation, we were unable to formally determine the sensitivity and specificity of the methods to detect these mutations, as compared with wild-type *EGFR*, in serum or plasma. Further, the different sample media used in each study (plasma *vs* serum) prevented combining the study data. Although combination of the LL3 and LL6 data would have potentially improved the overall statistical power of the outcomes analyses, the separate analysis of plasma and serum did provide a preliminary assessment of which media may be better for the detection of *EGFR* mutations. As described in the Methods section, all analyses were *post hoc* and therefore considered exploratory. No adjustment for multiplicity was made to the *P* values presented, resulting in an increased chance of false-positive results; however, the results were generally consistent and of sufficient magnitude to suggest a true effect rather than a chance finding. Finally, serial postbaseline blood sampling was not conducted in LL3 and LL6; thus potential changes in blood mutation status during treatment and at the time of progression could not be assessed.

In summary, our findings suggest that detection of *EGFR* mutation in cfDNA from plasma appears to be a promising and minimally invasive alternative to tumour biopsy for patients without available tissue samples. Patients with *EGFR* mutations detected in the blood (irrespective of mutation type) displayed more advanced disease characteristics and had worse prognosis compared with blood *EGFR* mutation-negative patients. Importantly, clinical benefit with afatinib over platinum-based chemotherapy was consistently observed in both cfDNA+ and cfDNA− populations. Improvements in the methodology of cfDNA testing for *EGFR* mutations are needed to increase sensitivity and to enable this to be offered to all patients considered for EGFR-targeted therapy, with the goal of reducing the overall burden of performing invasive biopsy procedures. Future prospective studies in patients with paired tumour tissue and blood samples will be important to further develop the utility of this methodology with regard to patient prognosis and predicting clinical outcomes.

## Figures and Tables

**Table 1:** Detection of individual *EGFR* mutations between paired blood-derived cfDNA samples^a^ **and tumour tissue samples** b

	**Tumour tissue/cfDNA**								
**Mutation type**	**+/+**	**+/−**	**−/−**	**−/+**	**cfDNA no result**	**Sensitivity (%)**	**Specificity (%)**	**PPV (%)**	**NPV (%)**
**LUX-Lung 3**									
Del19^c^	49	98	131	4	5	33.3	97.0	92.5	57.2
L858R^c^	26	93	160	2	6	21.8	98.8	92.9	63.2
Exon20^c^	0	5	272	1	9	0.0	99.6	0.0	98.2
G719X^c^	0	5	272	0	10	0.0	100.0	–	98.2
L861Q^c^	1	5	270	1	10	16.7	99.6	50.0	98.2
S768I^c^	1	3	273	0	10	25.0	100.0	100.0	98.9
T790M^c^	3	7	264	1	12	30.0	99.6	75.0	97.4
Overall concordance^d^	82	205							
%**d**	**28.60**								
**LUX-Lung 6**									
Del19^c^	112	63	149	4	6	64.0	97.4	96.6	70.3
L858R^c^	70	57	200	1	6	55.1	99.5	98.6	77.8
Exon20^c^	5	2	321	0	6	71.4	100.0	100.0	99.4
G719X^c^	5	13	310	0	6	27.8	100.0	100.0	96.0
L861Q^c^	8	3	317	0	6	72.7	100.0	100.0	99.1
S768I^c^	3	4	321	0	6	42.9	100.0	100.0	98.8
T790M^c^	0	1	326	1	6	0	99.7	0.0	99.7
Overall concordance^d^	202	132							
%**d**	**60.50**								

Abbreviations: cfDNA=cell-free DNA; *EGFR*=epidermal growth factor receptor; NPV=negative predictive value; PPV=positive predictive value.

a Serum in LUX-Lung 3 and plasma in LUX-Lung 6.

b No patients included in this analysis were *EGFR* mutation negative; all patients had tumour tissue that was positive for one or more mutation type. The calculations for sensitivity, specificity, PPV and NPV make use of test results for all seven mutation types within each individual patient.

c Allele-level concordance.

d Overall (patient level) concordance of cfDNA mutation status compared with tumour tissue.

**Table 2:** Baseline and disease characteristics according to the presence of *EGFR* mutation by cfDNA analysis

	**LUX-Lung 3**			**LUX-Lung 6**		
**Characteristics**	**Serum cfDNA+ (*****n*****=82)**	**Serum cfDNA**− (***n*****=205)**	**Association OR (95% CI);** ***P*** **value**^a^	**Plasma cfDNA+ (*****n*****=202)**	**Plasma cfDNA**− (***n*****=132)**	**Association OR (95% CI);** ***P*** **value**^a^
**Gender,** ***n*** **(%)**						
Male	27 (32.9)	72 (35.1)	1.10 (0.64–1.90); 0.7238	63 (31.2)	57 (43.2)	1.68 (1.06–2.64); 0.0260
Female	55 (67.1)	133 (64.9)		139 (68.8)	75 (56.8)	
**Age, years**						
Median (range)	61 (38–83)	61 (28–84)	0.94 (0.73–1.22); 0.6487	58 (29–77)	58 (27–78)	1.00 (0.82–1.22); 0.9683
**Race,** ***n*** **(%)**						
Asian	58 (70.7)	149 (72.7)	1.10 (0.63–1.94); 0.7391	202 (100.0)	132 (100.0)	–
Non-Asian	24 (29.3)	56 (27.3)		0	0	
**ECOG PS,** ***n*** **(%)**						
0	17 (20.7)	81 (39.5)	2.50 (1.37–4.56); 0.0029	36 (17.8)	43 (32.6)	2.23 (1.34–3.72); 0.0022
1	65 (79.3)	124 (60.5)^b^		166 (82.2)	89 (67.4)	
**Smoking status, *n* (%)**						
Current	2 (2.4)	4 (2.0)		16 (7.9)	10 (7.6)	
Former	23 (28.0)	62 (30.2)	1.08 (0.62–1.88); 0.7789^c^	26 (12.9)	28 (21.2)	1.54 (0.93–2.56); 0.0953^c^
Never	57 (69.5)	139 (67.8)		160 (79.2)	94 (71.2)	
**Adenocarcinoma stage, *n* (%)**						
IIIB with pleural effusion	7 (8.5)	24 (11.7)	1.42 (0.59–3.44); 0.4364	7 (3.5)	10 (7.6)	2.28 (0.85–6.16); 0.1029
IV	75 (91.5)	181 (88.3)		195 (96.5)	122 (92.4)	
**Differentiation grade, *n* (%)**						
Well differentiated	3 (3.7)	27 (13.2)	1.23 (0.67–2.26)^d^; 1.77 (0.87–3.60)^e^; 0.2825	10 (5.0)	15 (11.4)	1.56 (0.92–2.65)^d^; 2.23 (1.27–5.03)^e^; 0.0295
Moderately differentiated	19 (23.2)	42 (20.5)		30 (14.9)	25 (18.9)	
Poorly differentiated	16 (19.5)	28 (13.7)		46 (22.8)	17 (12.9)	
Undifferentiated	6 (7.3)	11 (5.4)		2 (1.0)	2 (1.5)	
Not specified	38 (46.3)	97 (47.3)		114 (56.4)	73 (55.3)	
**Number of metastatic sites,** ***n*** **(%)**						
0	0 (0.0)	6 (2.9)	1.81 (0.91–4.07)^f^; 3.94 (1.94–7.98)^g^; 0.0003	1 (0.5)	3 (2.3)	2.06 (1.22–3.48)^f^; 4.20 (2.32–7.61)^g^; <0.0001
1	12 (14.6)	64 (31.2)		50 (24.8)	61 (46.2)	
2	18 (22.0)	58 (28.3)		74 (36.6)	45 (34.1)	
⩾3	52 (63.4)	77 (37.6)		77 (38.1)	23 (17.4)	
**Location of metastatic sites,** ***n*** **(%)**						
Pleural effusion	43 (52.4)	82 (40.0)	1.65 (0.99–2.77); 0.0558	63 (31.2)	42 (31.8)	0.97 (0.61–1.56); 0.9034
Bone	54 (65.9)	76 (37.1)	3.27 (1.91–5.60); <0.0001	119 (58.9)	28 (21.2)	5.33 (3.22–9.80); <0.0001
Brain	13 (15.9)	27 (13.2)	1.24 (0.61–2.55); 0.5538	24 (11.9)	22 (16.7)	0.67 (0.36–1.26); 0.2166
Liver	25 (30.5)	18 (8.8)	4.56 (2.32–8.95); <0.0001	37 (18.3)	4 (3.0)	7.18 (2.49–20.65); 0.0003
Other	60 (73.2)	142 (69.3)	–	164 (81.2)	101 (76.5)	–
Median (range) SLD target lesions, mm	60.5 (14.1–168.5)	45.6 (10.6–167.0)	1.14 (0.99–1.31); 0.0613	56.8 (16.1–175.8)	46.2 (12.0–162.6)	1.35 (1.16–1.58); 0.0001
**Tissue** ***EGFR*** **mutation type,** ***n*** **(%)**						
Common mutations	75 (91.5)	183 (89.3)	0.59 (0.34–1.02)^h^; 0.62(0.25–1.56)^i^; 0.1427	184 (91.1)	114 (86.4)	0.73 (0.46–1.17)^h^; 0.54 (0.26–1.12)^i^; 0.1732
Del19	49 (59.8)	96 (46.8)		111 (55.0)	60 (45.5)	
L858R	26 (31.7)	87 (42.4)		73 (36.1)	54 (40.9)	
Uncommon mutations^j^	7 (8.5)	22 (10.7)		18 (8.9)	18 (13.6)	
**Laboratory parameters, median (range)**						
WBC count, 10^9^ l^−1^	7.8 (3.2–29.9)	7.0 (2.4–24.4)	1.09 (1.02–1.17); 0.0132	7.4 (2.9–18.6)	6.8 (3.5–24.1)	1.08 (1.00–1.17); 0.0599
LDH, U l^−1^	287 (125–1706)	245 (93–1160)	1.06 (1.02–1.11); 0.0023	224 (89–1773)	170 (54–431)	1.16 (1.06–1.26); <0.0001
ALP, U l^−1^	124 (40–973)	125 (36–513)	1.10 (1.04–1.17); 0.0025	98 (31–2463)	83 (39–379)	1.49 (1.28–1.74); 0.0006

Abbreviations: ALP=alkaline phosphatase; cfDNA=cell-free DNA; CI=confidence interval; ECOG PS=Eastern Cooperative Oncology Group performance status; *EGFR*=epidermal growth factor receptor; LDH=lactate dehydrogenase; OR=odds ratio; SLD=sum of longest diameter; WBC=white blood cell.

a Two-sided Wald chi-square test from univariate logistic regression analysis.

b Includes one patient with an ECOG PS of 2.

c Never *vs* former/current smoker.

d Not specified *vs* well/moderately differentiated.

e Well/moderately differentiated *vs* poorly/undifferentiated.

f 0/1 *vs* 2 metastatic sites.

g 0/1 *vs* ⩾3 metastatic sites.

h Del19 *vs* L858R mutations.

i Del19 *vs* other mutations.

j Including T790M, Ins20, G719X, S768I, and L861Q, alone or as complex mutations in two or more exons.

**Table 3:** PFS and OS according to the presence of an *EGFR* mutation by cfDNA analysis^a^ **in patients with common *EGFR* mutations (Del19 or L858R; based on tumour biopsy)**

	**cfDNA+**		**cfDNA−**			
**LUX-Lung 3**	**Afatinib**	**Cisplatin+pemetrexed**	**Afatinib**	**Cisplatin+pemetrexed**	**Effect of cfDNA result;** ***P*** **value**	**Interaction between treatment and blood test results;** ***P*** **value**
**Tissue: common *EGFR* mutation (Del19 or L858R)**						
Patients, *n*	50	25	121	62		
Median PFS, months	8.3	3.3	13.7	6.9		
Afatinib *vs* CT, HR (95% CI); *P* value	**0.35 (0.18–0.67) 0.0009**		**0.46 (0.31–0.68) <0.0001**		0.0037	0.1242
Median OS, months	22.1	14.7	33.6	28.6		
Afatinib *vs* CT, HR (95% CI) *P* value	**0.64 (0.37–1.11) 0.1062**		**0.75 (0.50–1.12) 0.1578**		0.0003	0.5353
**Tissue: Del19**						
Patients, *n*	30	19	67	29		
Median PFS, months	8.2	3.3	16.4	5.8		
Afatinib *vs* CT, HR (95% CI) *P* value	**0.30 (0.13–0.67) 0.0017**		**0.17 (0.09–0.33) <0.0001**		0.0382	0.9608
Median OS, months	29.4	14.3	33.3	28.2		
Afatinib *vs* CT, HR (95% CI) *P* value	0.50 (0.26–0.97) 0.0372		0.54 (0.32–0.93) 0.0247		0.0355	0.7512
**Tissue: L858R**						
Patients, *n*	20	6	54	33		
Median PFS, months	7.6	2.8	13.6	7.4		
Afatinib *vs* CT, HR (95% CI) *P* value	0.40 (0.11–1.50) 0.1593		0.78 (0.46–1.35) 0.3698		0.2097	0.1376
Median OS, months	16.5	14.8	33.9	29.5		
Afatinib *vs* CT, HR (95% CI) *P* value	1.12 (0.37–3.40) 0.8422		1.08 (0.59–1.97) 0.8071		0.0086	0.9247
**LUX-Lung 6**	**Afatinib**	**Cisplatin+gemcitabine**	**Afatinib**	**Cisplatin+gemcitabine**	**Effect of cfDNA result;** ***P*** **value**	**Interaction between treatment and blood test results;** ***P*** **value**
**Tissue: common *EGFR* mutation (Del19 or L858R)**						
Patients, *n*	135	49	72	42		
Median PFS, months	9.7	4.6	16.6	5.8		
Afatinib *vs* CT, HR (95% CI) *P* value	0.25 (0.16–0.39) <0.0001		0.12 (0.06–0.24) <0.0001		<0.0001	0.4057
Median OS, months	20	17.8	35.6	27		
Afatinib *vs* CT, HR (95% CI) *P* value	0.78 (0.54–1.13) 0.1841		0.59 (0.36–0.98) 0.0383		<0.0001	0.4405
**Tissue: Del19**						
Patients, *n*	80	31	41	19		
Median PFS, months	11	4.5	16.6	6.9		
Afatinib *vs* CT, HR (95% CI) *P* value	0.19 (0.10–0.34) <0.0001		0.10 (0.03–0.28) <0.0001		0.0135	0.7958
Median OS, months	23.6	14.6	39.4	21.1		
Afatinib *vs* CT, HR (95% CI) *P* value	0.67 (0.41–1.09) 0.106		0.37 (0.18–0.78) 0.0063		0.0008	0.2101
**Tissue: L858R**						
Patients, *n*	55	18	31	23		
Median PFS, months	8.1	4.6	16.5	5.6		
Afatinib *vs* CT, HR (95% CI) *P* value	0.37 (0.18–0.74) 0.0032		0.17 (0.07–0.44) <0.0001		0.0022	0.2846
Median OS, months	17	17.9	27.7	28.2		
Afatinib *vs* CT, HR (95% CI) *P* value	0.96 (0.54–1.69) 0.8781		1.10 (0.55–2.21) 0.779		<0.0001	0.7792

Abbreviations: cfDNA=cell-free DNA; CI=confidence interval; CT=chemotherapy; *EGFR*=epidermal growth factor receptor; HR=hazard ratio; OS=overall survival; PFS=progression-free survival.

a Patients who were *EGFR* Del19 or L858R mutation positive based on tissue biopsy were grouped according to whether any *EGFR* mutation was detected by cfDNA analysis (cfDNA+ or cfDNA−).
