# Supplementary material for: EGFR mutation detection in circulating cell-free DNA of lung adenocarcinoma patients: analysis of LUX-Lung 3 and 6
Source: Br J Cancer. 2016 Dec 22;116(2):175–85. doi: 10.1038/bjc.2016.420 (PMC5243999; doi:10.1038/bjc.2016.420)
Supplement: Supplementary Figures [file bjc2016420x1.docx]

Supplementary Figure 1. Kaplan–Meier curves of PFS within treatment arms according to presence of an *EGFR* mutation by cfDNA analysis^a^ in patients with common *EGFR* mutations (Del19 or L858R; based on tumour biopsy) in (A) LL3 and (B) LL6.


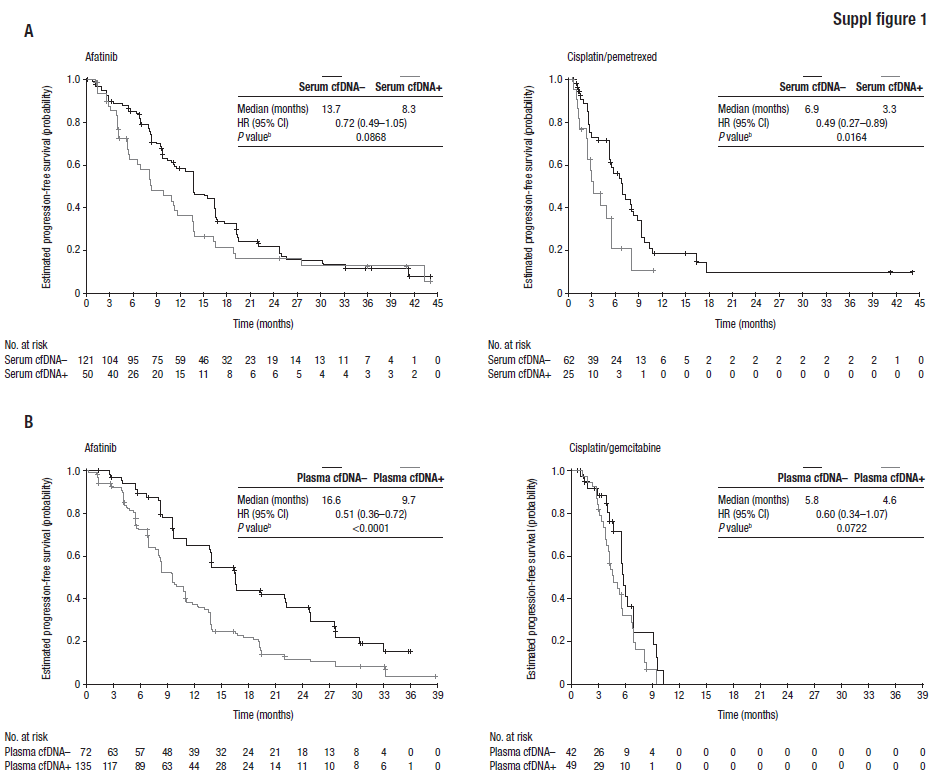


^a^Patients who were *EGFR* Del19 or L858R mutation-positive based on tissue biopsy were grouped according to whether any *EGFR* mutation was detected by cfDNA analysis (cfDNA+ or cfDNA–).

^b^Log-rank test.

### Supplementary Figure 2. Kaplan–Meier curves of OS within treatment arms according to presence of an EGFR mutation by cfDNA analysis^a^ in patients with common EGFR mutations (Del19 or L858R; based on tumour biopsy) in (**A**) LL3 and (**B**) LL6.


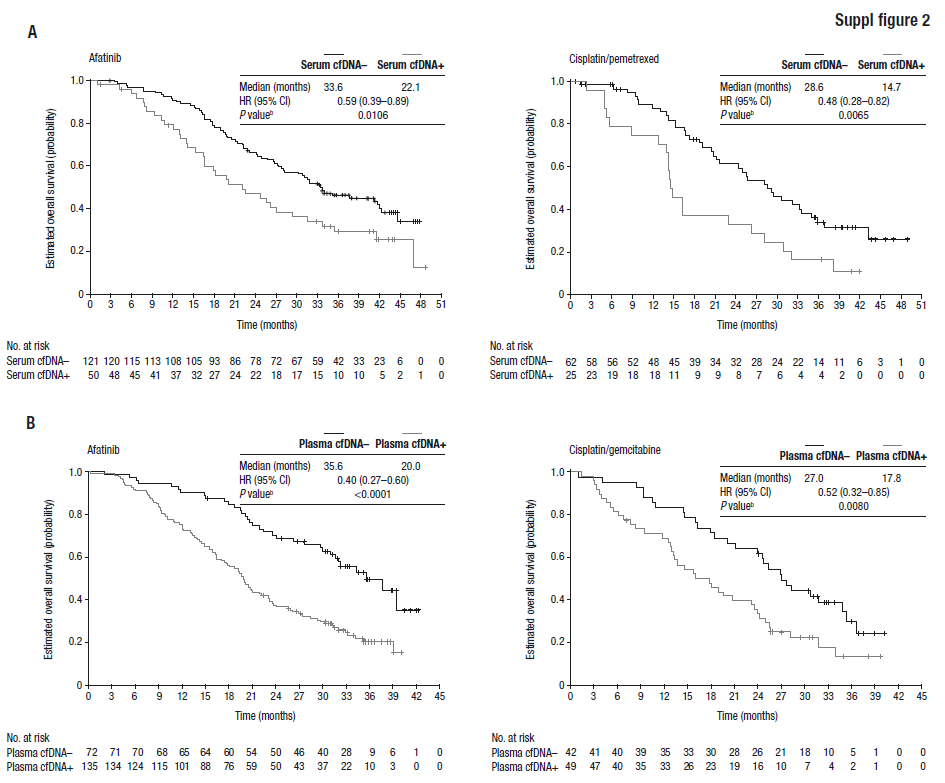


^a^Patients who were *EGFR* Del19 or L858R mutation-positive based on tissue biopsy were grouped according to whether any *EGFR* mutation was detected by cfDNA analysis (cfDNA+ or cfDNA–).

^b^Log-rank test.
